# Vaccination with ancestral SARS-CoV-2 spike adjuvanted with TLR agonists provides cross-protection against XBB.1

**DOI:** 10.1038/s44298-024-00038-0

**Published:** 2024-08-06

**Authors:** Stephanie K. Lathrop, Jordan J. Clark, Karthik Siram, Robert Andreata-Santos, Jeremy Yong, Rebekah D. Tee, Clara J. Davison, Gagandeep Singh, David Burkhart, Florian Krammer, Jay T. Evans

**Affiliations:** 1 Center for Translational Medicine, Department of Biomedical and Pharmaceutical Sciences, School of Pharmacy, University of Montana, Missoula, MT USA; 2 Department of Microbiology, Icahn School of Medicine at Mount Sinai, New York, NY USA; 3 Center for Vaccine Research and Pandemic Preparedness (C-VaRPP), Icahn School of Medicine at Mount Sinai, New York, NY USA; 4 Department of Pathology, Molecular and Cell Based Medicine, Icahn School of Medicine at Mount Sinai, New York, NY USA; 5 Retrovirology Laboratory, Department of Microbiology, Immunology and Parasitology, Paulista School of Medicine, Federal University of São Paulo (UNIFESP), São Paulo, SP Brazil; 6 Ignaz Semmelweis Institute, Interuniversity Institute for Infection Research, Medical University of Vienna, Vienna, Austria

**Keywords:** Vaccines, Virology, Adaptive immunity, Translational immunology, Vaccines

## Abstract

Many different platforms have been used to develop highly protective vaccines against severe acute respiratory syndrome coronavirus 2 (SARS-CoV-2) in humans. However, protection has eroded over time due to the emergence of antigenically diverse viral variants, especially the Omicron subvariants. One successful platform for the generation of SARS-CoV-2 vaccines are recombinant spike protein vaccines, of which two are licensed in the United States and Europe. Typically, purified recombinant protein antigens are poorly immunogenic and adjuvants must be included in the formulation. Here, we adjuvanted recombinant ancestral SARS-CoV-2 Wuhan-Hu-1 spike proteins with an emulsion formulation combined with synthetic Toll-like receptor (TLR) 4 and 7/8 agonists. This combination led to the induction of a Th1-skewed immune response that included high titers of antibodies against Wuhan-Hu-1 spike. These serum antibodies included neutralizing and cross-reactive antibodies that recognized the spike from multiple SARS-CoV-2 variants, as well as the receptor binding domain (RBD) from SARS-CoV-1. Despite an absence of robust cross-neutralization, vaccination against Wuhan-Hu-1 spike in the context of TLR-containing emulsions provided complete cross-protection against disease from a lethal challenge with XBB.1 in a stringent K18-hACE2 mouse model. We believe that the combination of recombinant spike antigens with TLR agonist-based emulsion formulations could lead to the development of next-generation SARS-CoV-2 vaccines that provide significant protection from future emerging variants.

## Introduction

Severe acute respiratory syndrome coronavirus 2 (SARS-CoV-2) vaccines have been invaluable in preventing severe disease and death^1,2^ in the nearly three years since they were approved for emergency use in the US by the FDA^1^. Two of the three vaccines currently available in the US are the mRNA-based, lipid nanoparticle-formulated vaccines produced by Pfizer/BioNTech (BNT162b2) and Moderna (mRNA-1273). The downside of these vaccines is their requirement for ultra-cold storage, which increases cost and decreases availability, and their novelty which, along with a rapid approval process, may have resulted in lower rates of public confidence. Adoption of the previously-available Wuhan-Hu-1/BA.5 bivalent booster in the US only reached an estimated 17% of eligible residents^3^. In addition, studies suggest that the level of neutralizing antibodies is not maintained against novel variants of concern as would be desirable, further increasing the need for boosting^4^.

The third vaccine now available, produced by Novavax (Nuvaxovid), is a baculovirus-produced recombinant protein-based vaccine. Such vaccines (like the influenza vaccine Flublok) have a longer history of safety and do not require special storage conditions. However, the absence of endogenous immunostimulatory signals in purified proteins necessitates the addition of adjuvants to these formulations to direct the body to create the needed immune response. The Novavax vaccine includes a saponin-based adjuvant which requires the harvest and purification of natural saponins from the *Q. saponaria* tree^5^, a potential manufacturing hurdle.

Another successful class of adjuvants is the squalene-based emulsions, two of which, AS03™ (GSK) and MF59™ (Novartis), are approved for use in pandemic influenza vaccines. Emulsions have been shown to elicit a large boost in neutralizing antibody titers^6^; however, the constant emergence of new SARS-CoV-2 variants that escape antibody neutralization requires that vaccines which rely on eliciting neutralizing antibodies will need to be continually updated. We hypothesize that the additional development of a strong T cell-mediated response will provide enhanced protection against variants that have escaped antibody recognition, since most T cell epitopes will remain conserved even as key antigenic sites are mutated.

Previously, we have demonstrated that the combination of a synthetic Toll-like receptor (TLR)7/8 and/or TLR4 agonist with an AS03-like emulsion caused a strong shift away from T helper cell type 2 (Th2)-mediated immunity, instead encouraging the development of a Th1/Th17 dominated response, while still retaining the superior antibody titers characteristic of an emulsion adjuvant^7^. While that study used recombinant receptor binding domain (RBD) of spike as antigen, we show here that the recombinant, trimerized, spike ectodomain is a more inherently immunogenic antigen which engages a greater T cell response, perhaps because it likely contains conserved T-cell epitopes not present in the much smaller RBD. In these studies, we utilized the ancestral SARS-CoV-2 spike trimer derived from Wuhan-Hu-1 adjuvanted with AS03-like or MF59-like emulsions and examined the impact of including novel synthetic TLR7/8 and/or TLR4 agonists in these emulsions. We show that the inclusion of a TLR agonist enhances cross-protection against disease from the highly mutated Omicron XBB.1 variant in mice.

## Results

### Antigen selection

Previously, our group reported vaccination with TLR agonist adjuvanted AS03-like emulsion formulations using the purified RBD of SARS-CoV-2 as antigen^7^. While there are distinct advantages for using RBD, such as ease of antigen production, we suspected that using a stabilized trimer of the spike ectodomain might improve immunogenicity. Pilot experiments testing the immune response after vaccination with multiple doses of the spike trimer (henceforth referred to as spike) or the spike RBD demonstrated that 1 μg of spike and 3 μg of RBD were adequate to induce appreciable antibody titers against each antigen (data not shown). We then directly compared the results of immunization with 1 μg spike to 3 μg RBD with and without adjuvantation by either 10 μg of the TLR7/8 agonist INI-4001 or 10 μg of the TLR4 agonist INI-2002 in aqueous formulations. Serum antibody titers against RBD were higher after vaccination with 1 μg of spike than with 3 μg of RBD, whether adjuvanted or not, even though this dose contains approximately 17 times more RBD (by molar equivalents) than is provided by 1 μg of spike (Supplementary Fig. 1A). We suspect that the larger size and multimeric properties of the trimerized spike protein contribute to a more efficient recognition and response by the immune system. We also noted that addition of INI-4001 significantly boosted the anti-RBD IgG titers with spike but not with RBD, while INI-2002 adjuvanted both antigens well.

Cells isolated from the draining lymph nodes (dLN) 21 days after the booster vaccination were cultured with whole spike protein for approximately 72 h, and the cytokine profile of the supernatants was examined via multiplex enzyme-linked immunosorbant assay (ELISA). Overall, the level of cytokines, assumed to be reflective of the number of antigen-specific T cells in the cultures, were higher from mice vaccinated with spike antigen than with RBD (Supplementary Fig. 1B). This makes sense when considering the larger assortment of epitopes available to activate T cells from the full-length spike protein. Induction of the Th2 cytokine interleukin (IL)-5 was significantly higher in mice vaccinated with 1 μg spike when compared to 3 μg of RBD, while the addition of TLR agonist INI-4001 or INI-2002 significantly suppressed the development of IL-5 producing cells, instead enhancing interferon (IFN)γ and IL-17A production (indicative of a Th1 and Th17 response, respectively). Considering these results, we selected the spike trimer as the more effective antigen for use in subsequent vaccine studies.

### Formulation of squalene-based emulsions with TLR agonists

Previous studies demonstrated that adjuvanting the RBD antigen with squalene-based emulsions provided significantly improved antibody titers and T cell responses, despite this antigen’s overall lower immunogenicity^7^. These studies further showed that addition of INI-4001 and/or INI-2002 in the emulsions further enhanced antibody titers and T cell development, and strongly skewed the response away from a Th2 and toward a Th1-dominated one (confirmed in Supplementary Fig. 1B). As we seek to induce both high neutralizing antibody titers and a strong anti-viral T cell responses, with the hypothesis that a strong Th1 response would enhance protection against a heterologous challenge, we chose to use a similar set of emulsion-based adjuvants in conjunction with the spike trimer antigen in the vaccine studies presented here.

**Fig. 1:**
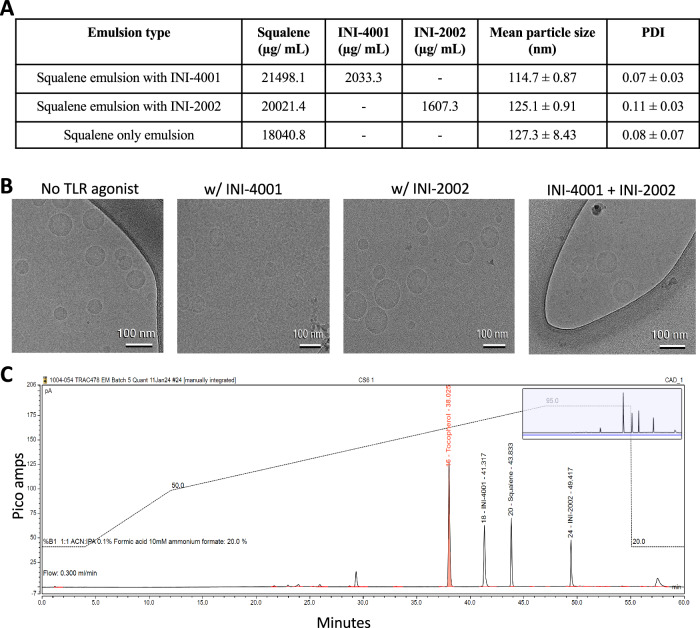
Characterization of AS03-like emulsions, with and without TLR agonists. **A** The concentrations of squalene, INI-2002, and INI-4001 in each starting emulsion created for these studies, and the mean particle size and polydispersity index for each. **B** Cryo-TEM images of each emulsion shown in (**A**), plus the resulting emulsion after mixing of the INI-4001 and INI-2002 emulsions. **C** Example UHPLC chromatograph showing the distinct peaks for INI-2002, INI-4001, DL-α-tocopherol, and squalene.

The emulsion adjuvant system used in this study contains two biodegradable oils (squalene and DL-α-tocopherol), with a surfactant (polysorbate-80) in the same ratio as AS03™ adjuvant, and in some formulations one or both TLR agonists (INI-4001 and INI-2002). Consistent with our previously published data^7^, the emulsion prepared with INI-4001 had a relatively lower droplet size than the emulsions prepared with INI-2002 or without either agonist (Fig. 1A). This reduction in the particle size was attributed to the surfactant-like properties of the amphiphilic INI-4001. The low polydispersity index (PDI) indicated that the emulsion droplets were homogeneous, and therefore the methodology was efficient for producing homogeneous emulsions. Morphological evaluation of the emulsion droplets using a cryo-TEM (Fig. 1B) revealed spherical, uniform, unaggregated particles, even after mixing the INI-4001 and INI-2002 emulsions (far right), suggesting that admixing did not lead to instability. An ultra high performance liquid chromatography (UHPLC) method was tested for its applicability in the quantitation of the emulsion components INI-4001, INI-2002, DL-α-tocopherol, and squalene. Intermediate precision (<%5 relative standard deviation, *n* = 14) and regression models (R2 ≥ 0.999) over the assay concentration range were confirmed using reference standards. The assay demonstrated the formulation process to be reproducible, and recovery of the assayed components in formulation samples were consistently ±10% for the TLR agonists and ±15% for the excipients. A typical chromatogram showing the separation of INI-4001, INI-2002, DL-α-tocopherol, and squalene is shown in Fig. 1C. Based on the concentrations of INI-4001 and INI-2002 in the emulsion, the appropriate amount to provide 10 μg of INI-4001 and/or INI-2002 per dose was mixed with the antigen to make the final vaccines. Of note, the final squalene dose was adjusted to 180 μg per vaccination for all groups. This represents a lower dose than is recommended by the manufacturer for AddaS03™and Addavax™ (1.07 mg squalene).

### TLR agonists in emulsions skew the adaptive immune response

**Fig. 2:**
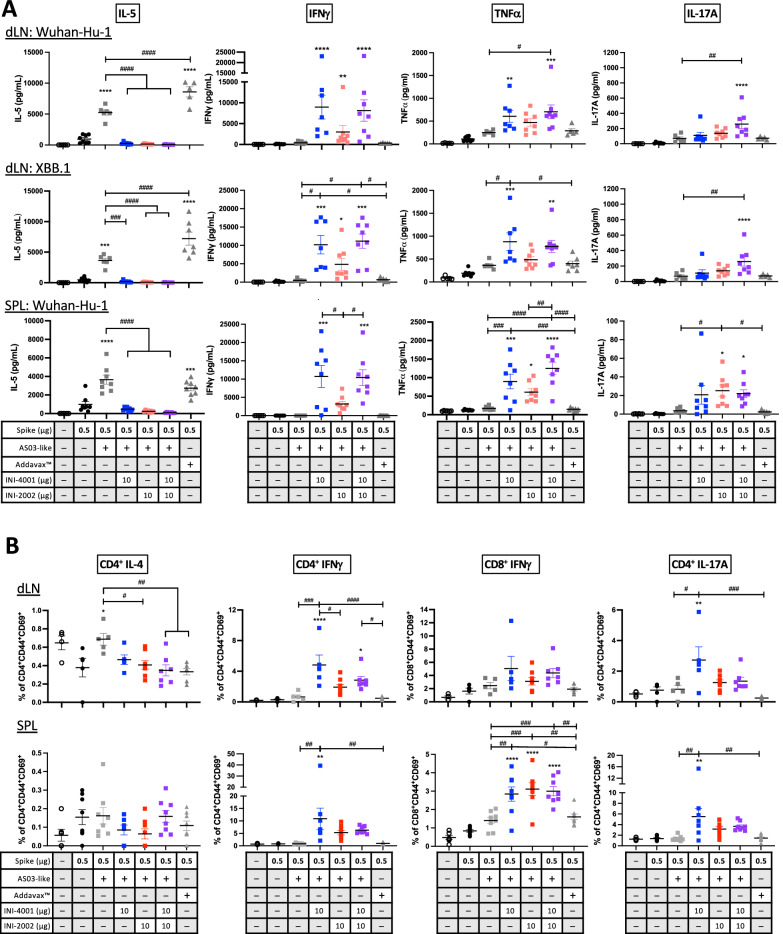
Including TLR agonists in emulsion-based adjuvants promotes development of Th1 and Th17 cells and inhibits Th2. **A** Cells from draining LNs (dLN) or spleen (SPL) isolated 21 days after booster vaccination were cultured with Wuhan-Hu-1 or XBB.1 spike for 72 h. Culture supernatants were assayed for cytokines by multiplex ELISA. **B** The same cell populations were cultured with Wuhan-Hu-1 spike protein and pooled overlapping spike peptides for 18 h, then analyzed for T cell cytokine production by flow cytometry. Differences between groups were analyzed by one-way ANOVA with correction for multiple comparisons by Tukey post-test. Asterisks denote significant differences versus unadjuvanted Spike antigen and # indicate differences between other groups. *^/#^*p* < 0.05, ^##^*p* < 0.01, ^***/###^*p* < 0.001, ****^/####^*p* < 0.0001.

Mice were vaccinated two times at a three-week interval, and twenty-one days after the booster the extent and nature of the T cell response was assessed by collection of the draining LNs and the spleen. These cells were dissociated and cultured with recombinant spike protein derived from Wuhan-Hu-1 or Omicron XBB.1 for approximately 72 hours, after which the culture supernatant was collected and tested for cytokines. Additional cells were cultured with both Wuhan-Hu-1 spike protein and an overlapping peptide pool of the spike protein for 18 h, then stained for analysis by flow cytometry. Immunization with either the AS03-like emulsion or, for comparison, Addavax^™^ (a research-grade variation of the MF59 emulsion) resulted in the development of a Th2-dominated immune response, as evidenced by release of the Th2-associated cytokine IL-5 upon restimulation (Fig. 2A). However, when TLR7/8 agonist INI-4001 or TLR4 agonist INI-2002 was added to the emulsion, the production of IL-5 was significantly reduced, and higher levels of IFNγ and tumor necrosis factor (TNF)α were observed, indicative of a skewing of the cell-mediated response towards a Th1 phenotype. Notably, these results were similar whether restimulating with Wuhan-Hu-1 or XBB.1 antigen, suggesting the response was largely in response to conserved T cell epitopes. Production of IL-17A was also detected in these groups, particularly when both TLR agonists were combined, suggesting that a mix of both Th1 and Th17 cells was produced by addition of the TLR agonists. Flow cytometry lent support to these results by demonstrating a higher percentage of IL-4-producing CD4^+^ T cells in the AS03-like emulsions group, although an increase in IL-4-producing cells was not detected in the Addavax™ group, as might have been expected from the supernatant cytokines (Fig. 2B). The significantly higher percentage of IFNγ-producing CD4^+^ T cells from groups that received the TLR agonists mirrored the data obtained from the supernatant measurements, and a small (though not statistically significant) increase in IFNγ-producing CD8^+^ T cells was also detected. An increased percentage of IL-17A-producing cells was also noted in the INI-4001 adjuvanted group, but not in the INI-2002 or combination group, as was previously detected in the antigen-restimulated supernatants. Together, these data indicate that inclusion of TLR agonists in these emulsions significantly impacts the resulting cell-mediated immunity by skewing it away from a Th2 to a Th1-dominated response, especially in the presence of the TLR7/8 agonist INI-4001.

### TLR agonists in emulsions enhance humoral immunity

**Fig. 3:**
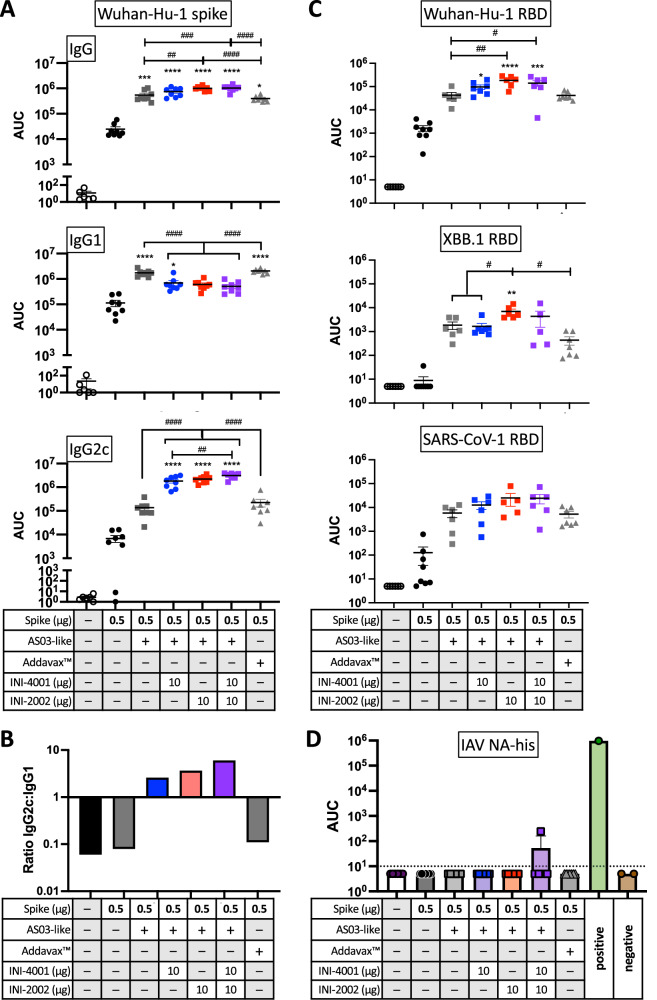
Addition of TLR agonists to emulsion adjuvants boosts the spike-specific antibody titers, increases the ratio of IgG2c to IgG1, and promotes cross-recognition of variants. **A** Serum antibody IgG titers against recombinant Wuhan-Hu-1 spike protein at 21 days after booster vaccination. **B** The mean AUC value for IgG2c was divided by that of IgG1 for each group. **C** Serum antibody IgG titers against recombinant spike RBD from the indicated virus. **D** Control ELISA against his-tagged neuraminidase (NA) from influenza A virus (IAV); dotted line indicates the limit of detection. Differences between groups were analyzed by one-way ANOVA with multiple comparisons by Tukey post-test. Asterisks denote significant differences versus unadjuvanted spike antigen and # indicate differences between other, indicated groups. *^/#^*p* < 0.05, ^##^*p* < 0.01, *^**/###^*p* < 0.001, ****^/####^*p* < 0.0001.

Measurement of the antigen-specific serum antibody titers 21 days after booster vaccination demonstrated the ability of emulsions to significantly improve spike-specific IgG development over that of the antigen alone (Fig. 3A, Supplementary Fig. 2). The addition of one or both TLR agonists further increased the titers and altered the relative amounts of IgG subtypes produced. The addition of INI-4001 or INI-2002 (alone or in combination) significantly increased the antigen-specific IgG2c levels while decreasing IgG1 suggesting a shift away from a Th2-dominated response towards a Th1 phenotype. Directly comparing the ratio of antigen-specific IgG2c:IgG1 further exemplifies the shift in humoral immunity by the addition of TLR agonists to the emulsion formulation (Fig. 3B).

Next, we evaluated whether the increased spike-specific antibody titers improved the recognition of the spike RBD from other variants. The RBD contains the highest concentration of mutations between variants, and binding in this region is effective in preventing infection by inhibiting viral entry. We therefore investigated the breadth of binding antibodies elicited by the different adjuvant formulations by assaying the binding of serum antibodies to the spike RBD of ancestral Wuhan-Hu-1 SARS-CoV-2, highly mutated Omicron XBB.1 SARS-CoV-2, and SARS-CoV-1^8^ (Fig. 3C). As the recombinant RBDs and the vaccination antigen were purified with the assistance of a His tag, a control ELISA against a His-tagged irrelevant antigen (influenza A neuraminidase) was also done, and showed no measureable binding (Fig. 3D). While the titer of serum antibodies binding to SARS-CoV-1 RBD was predictably lower than to Wuhan-Hu-1, by roughly 10-fold, the relative titers between the groups remained similar, suggesting that a consistent proportion of antibodies generated against Wuhan-Hu-1 were cross-reactive. Binding to XBB.1 RBD did not follow this trend; rather, it revealed differences between the groups in the extent of cross-reactivity, with the INI-2002-containing emulsion promoting significantly greater cross-reactivity to the XBB.1 RBD as compared to the INI-4001-containing emulsion and emulsions lacking a TLR agonist. The presence of INI-4001 appeared to slightly impair the develoment of these cross-reactive antibodies, as the group receiving both TLR agonists did not achieve the titer seen with INI-2002 only.

### Enhanced antibody cross-reactivity from TLR adjuvants

**Fig. 4:**
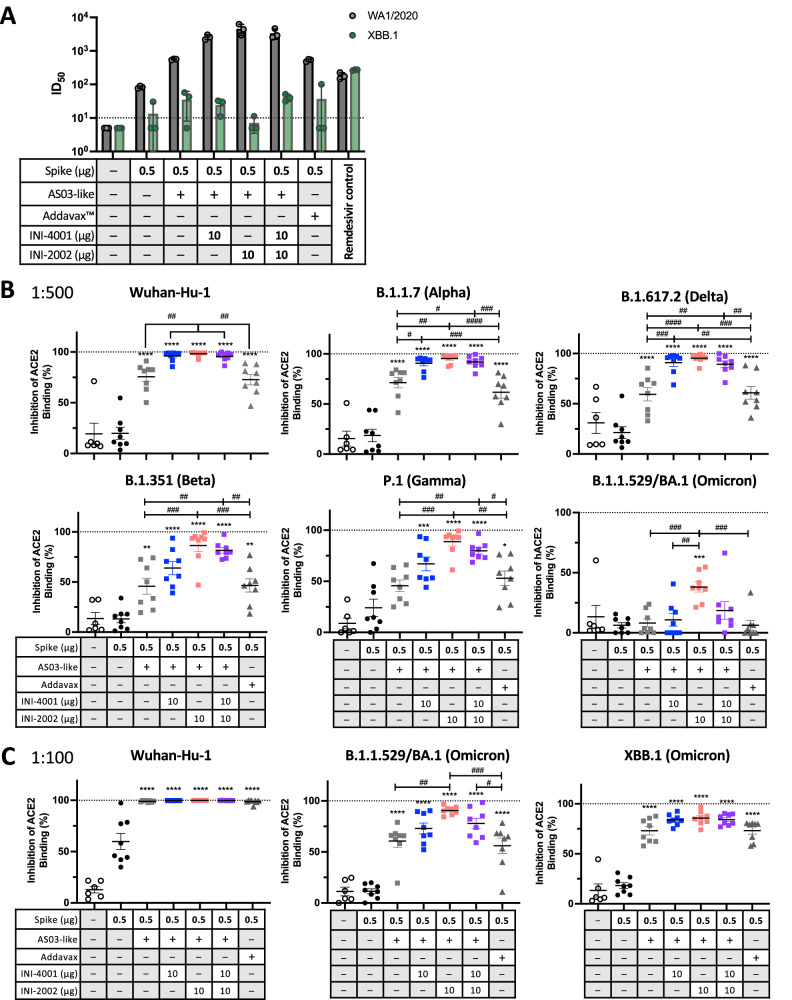
Neutralization potential of serum from vaccinated mice. **A** Serum collected 21 days after booster vaccination (day 42) was used in a microneutralization assay against strain WA1/2020 (gray) and Omicron XBB.1 (green). Anti-viral drug remdesivir was used as a positive control. Each symbol represents pooled sera from two animals. The dotted line represents the limit of detection. **B**, **C** Serum collected 21 days after booster vaccination (day 42) was tested for the ability to block the binding of soluble human ACE2 to recombinant spike trimers from the indicated strains by multiplex competitive ELISA. Serum was diluted 1:500 in (**B**), and 1:100 in (**C**). See also Supplementary Fig. 4 for data with additional Omicron variants. Differences between groups were analyzed by one-way ANOVA with multiple comparisons by Tukey post-test. Asterisks denote significant differences versus unadjuvanted spike antigen and #’s indicate differences between other indicated groups. *^/#^*p* < 0.05, ^##^*p* < 0.01, *^**/###^*p* < 0.001, ****^/####^*p* < 0.0001.

While the extent of binding to the RBD gives an indication of the ability of serum antibodies to prevent infection, a microneutralization assay more directly measures the prevention of viral entry and replication. The neutralizing titers of the sera against WA1/2020 closely reflected the total anti-spike IgG titers, as expected (Fig. 4A). However, neutralizing titers were reduced against XBB.1, with several of the samples unable to achieve a 50% reduction in infection at the highest serum concentration tested.

We further examined the cross-neutralizing potential of the serum antibodies by testing the serum in a spike-ACE2 interaction inhibition assay (SAIIA). In this assay, the ability of sera to prevent binding between immobilized recombinant spike trimers from several variants and soluble recombinant human ACE2 was measured (Fig. 4B, Supplementary Fig. 3A). The inhibition of binding between ACE2 and the Wuhan-Hu-1 strain largely mirrored the overall anti-spike serum IgG titers, in agreement with the microneutralization assay. The addition of TLR agonist INI-4001, INI-2002 or a combination of both TLRs significantly increased the binding inhibition across all spike variants. The sera inhibited binding to the B.1.1.7 (Alpha) and B.1.617.2 (Delta) spike proteins nearly as well as the Wuhan-Hu-1, but there was a notable loss against B.1.351 (Beta), P.1 (Gamma), and most especially Omicron BA.1. Differences among the emulsion vaccine groups in their ability to block ACE2 binding became more apparent when testing against the Omicron strains, in which a trend towards better neutralization potential from the TLR4 agonist containing emulsion became apparent. This trend mirrored the results of the ELISA against the Omicron XBB.1 RBD shown in Fig. 3C.

The loss of antibody-mediated ACE2 blocking against the Omicron variant BA.1 shown in the bottom right of Fig. 4B was undoubtedly due to the large number of mutations in the critical RBD region of this and other Omicron variants. This suggested that vaccination with Wuhan-Hu-1 spike protein would not provide a strong antibody-mediated neutralization response against this and possibly other Omicron strains. We further examined the ability of these sera to provide ACE2 binding inhibition against several Omicron variants (Fig. 4C, Supplementary Fig. 3B). In this assay, a higher concentration of serum (1:100 rather than 1:500) was used, due to the previously noted decrease in antibodies with inhibitory ability against BA.1 (Fig. 4B). At this dilution, differences between the adjuvanted groups in binding inhibition against Wuhan-Hu-1 were no longer detectable, but the ability of serum from TLR adjuvanted groups to better inhibit binding to BA.1, XBB.1, and other Omicron variants was revealed. These results verified that adjuvanting with TLR agonist-containing emulsions significantly improved the cross-reactivity of serum antibodies to several Omicron variants, with the emulsion containing INI-2002 providing a slightly better result (Fig. 4C, Supplementary Figs. 4 and 5). These levels of inhibition are obtained using a 5-fold greater concentration of serum, however, and therefore are still much lower than against the Alpha, Beta, Delta, and Gamma variants. In the context of the lower neutralizing antibody levels predicted for Omicron strains, we hypothesized that the strong T cell response noted in Fig. 2A may also contribute to protection in individuals vaccinated against an ancestral strain.

### Adjuvants enhance cross-protection against Omicron XBB.1

**Fig. 5:**
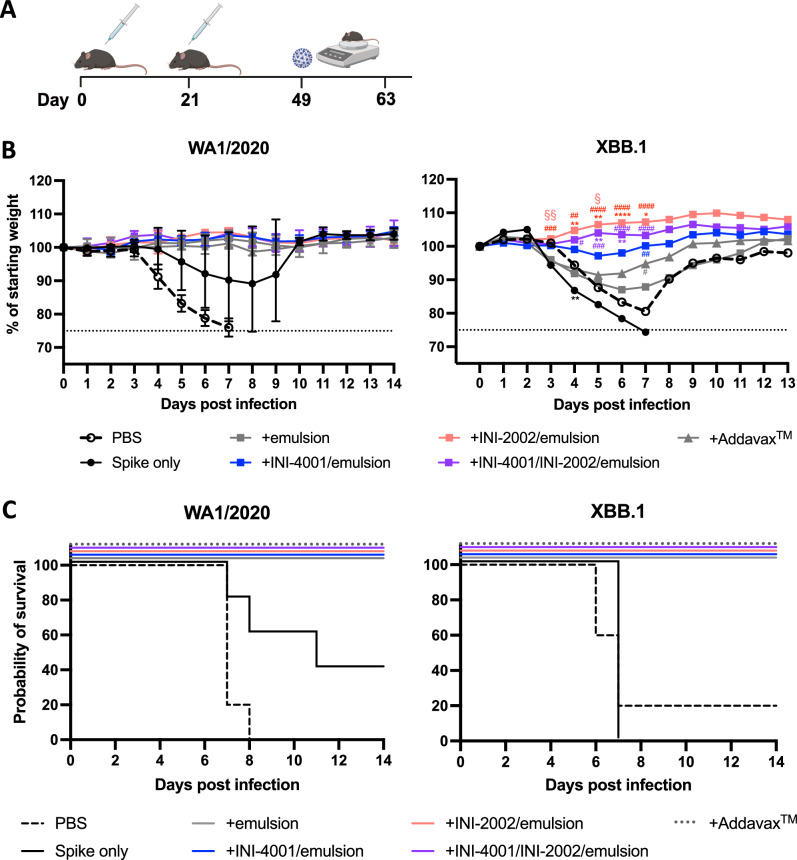
K18-hACE2 mice vaccinated with WA1/2020 spike adjuvanted with emulsions containing TLR agonists were better protected against weight loss after infection with Omicron variant XBB.1. **A** Schematic of experimental timeline (created with BioRender.com). **B** Weight change relative to starting weight (on day of infection), as recorded daily after infection with WA1/2020 (left) or XBB.1 (right). Dotted line indicates weight loss that requires humane euthanasia. Error bars in the WA1/2002 challenge represent the SEM; these were omitted from XBB.1 for clarity. Statistical significance determined by unpaired t-test with Welch correction at each time point, with Holm-Sidak multiple comparison correction. *indicates significance versus the PBS treated control, # versus the Spike only control, and § versus the emulsion group. *^/#/§^*p* < 0.05, **^/##/§§^*p* < 0.01^, ***/###^*p* < 0.001, ****^/####^*p* < 0.0001. **C** Survival of mice as a percentage of total mice in each group, recorded daily following infection.

To test whether the differences in antibody development and T cell responses elicited by this series of vaccines translated to differences in disease protection, K18-hACE2 mice were vaccinated with the Wuhan-Hu-1 spike protein, with or without the emulsion-based adjuvants, 21 days apart. Twenty-eight days after the booster vaccination the mice were infected with a 3xLD_50_ dose of either WA1/2020 or the Omicron strain XBB.1 via intranasal challenge (Fig. 5A). The weights of the mice were monitored for 14 days and any animals that lost ≥25% body weight were humanely euthanized.

Following WA1/2020 challenge, the groups receiving any of the emulsion-based adjuvants were completely protected from disease and did not exhibit any weight loss (Fig. 5B). Conversely, the unvaccinated group (receiving only the vehicle) succumbed to infection by 7 days post infection, and the group vaccinated with spike in the absence of adjuvant exhibited 40% survival (Fig. 5C). When challenged with the Omicron strain XBB.1, nearly all the animals receiving only the vehicle or unadjuvanted spike succumbed to the infection by day 7 post infection, except for one animal in the vehicle group that recovered. All animals that received an adjuvanted vaccine survived, but these groups exhibited varying levels of weight loss, demonstrating significant differences between the adjuvants in disease protection. The animals that received either Addavax™ or the AS03-like emulsion without additional TLR agonists exhibited the most weight loss but were able to recover. Strikingly, those vaccinated with TLR agonists showed greater protection from disease, with little to no weight loss over the course of the experiment. The level of weight loss correlated with the cross-reactive and potentially neutralizing antibody titers described in Figs. 3 and 4, with the INI-2002 adjuvanted animals faring the best and the combination INI-2002 + INI-4001 close behind, both exhibiting significantly less weight loss compared to the PBS and spike only groups between days 3 and 7 post-infection. The group adjuvanted with INI-4001 exhibited a slightly higher weight loss during these days that did not reach statistical significance until day 7. These results demonstrate that a subunit vaccine consisting of Wuhan-Hu-1 spike protein adjuvanted with an emulsion can provide protection from a lethal infection by the highly mutated variant XBB.1, and that the addition of a synthetic TLR4 agonist, INI-2002, can significantly enhance disease resistance.

## Discussion

With these studies, we have explored the value of combining emulsions with TLR agonists in adjuvanting subunit vaccines to extend the breadth of protection from antigenically distant variants. Previous studies have shown that squalene-based emulsions such as AS03 and MF59 can adjuvant a vaccine’s immune response by increasing antigen-specific IgG titers and promoting the development of CD4^+^ T cells^6,9^. It speaks to the effectiveness of emulsion-based adjuvants that a subunit vaccine based on recombinant spike trimers from B.1.351 (Beta) adjuvanted with AS03 was developed and approved for use in the EU in late 2022^10^. Despite using spike from B.1.351 (Beta), it demonstrated cross-neutralization against Omicron variants and even SARS-CoV-1 in non-human primates^11^. It also provided comparable protection against disease and hospitalization with Omicron variants as the Pfizer bivalent vaccine encoding spike from the ancestral and BA-5 Omicron strain in a trial in adults aged 75 and over^12^.

The AS03-like and MF59-like (Addavax™) emulsions in our studies also provided increased anti-spike IgG titers compared to the unadjuvanted antigen. Notably, the addition of INI-4001 (TLR7/8 agonist), INI-2002 (TLR4 agonist) or a combination of both TLR agonists further increased spike specific humoral and cell-mediated immunity, in agreement with previous studies using TLR agonist in emulsion formulations across a variety of antigens^7,9,13–15^. The development of a vaccine against a rapidly mutating pathogen requires a broad neutralizing antibody response across antigenic variants, and TLR adjuvants have demonstrated the ability to increase cross-reactivity of the antibody repertoire in other systems^16,17^. As expected, antibodies generated against spike protein from Wuhan-Hu-1 showed a considerable loss of neutralization potential against other SARS-CoV-2 variants. However, the addition of INI-2002 or INI-4001 adjuvants significantly enhanced the cross-reactivity to Alpha, Beta, Gamma, Delta and Omicron variants, albeit still at lower levels than Wuhan-Hu-1. Overall, the ability of serum antibodies to block the binding of ACE2 to the spike proteins from Alpha (B.1.1.7) and Delta (B.1.617.2) strains was similar to the Wuhan-Hu-1 spike. The loss of neutralization potential against the spike from Beta (B.1.351) and Gamma (P.1) strains was greater than noted for Alpha and Delta, while the Omicron variant titers dropped approximately ten-fold, as expected from the extent of mutations relative to the ancestral strain. This loss of overall affinity revealed an interesting difference between the serum antibodies from the TLR agonist groups. The group vaccinated with INI-2002 adjuvant produced significantly higher serum antibody titers and inhibition of ACE2 binding when compared to spike alone or spike with emulsion across every spike variant evaluated, including a diverse assortment of Omicron variants. INI-2002 adjuvanted groups fared slightly better against many of these variants than those from the INI-4001 group, although the differences were not significant. While INI-2002 also trended towards higher overall antigen-specific IgG titer, as seen by ELISA against Wuhan-Hu-1 spike antigen, it is also possible that INI-2002 contributed to improved cross-reactivity through the development of an expanded repertoire of antibodies compared to INI-4001. Further studies are in progress to explore the nature of these antibody repertoires and the impact of adjuvants on these responses.

The loss of ACE2 binding inhibition observed with the spike from Omicron subvariants was confirmed using a microneutralization assay utilizing Omicron XBB.1 virus, wherein the neutralizing titers of each of the adjuvant formulations was significantly reduced compared to WA1/2020. Despite the reduction in neutralizing titers against XBB.1, all vaccinated groups exhibited some degree of anti-XBB.1 RBD IgG titers, albeit 10-100-fold lower than anti-Wuhan-Hu-1 RBD titers, and the INI-2002 adjuvanted group was significantly higher than the emulsion controls. Interestingly, the breadth of cross-reactive antibodies elicited by vaccination also extended to SARS-CoV-1 RBD, indicating that the tested protein-based vaccines adjuvanted with TLR agonists are capable of eliciting cross-reactive antibodies against antigenically distinct sarbecoviruses. The group receiving spike adjuvanted with the INI-2002-containing emulsion exhibited significantly increased IgG titers against XBB.1 spike RBD, which mirrors the observed differences in ACE2 blocking of this and other Omicron subvariants. Despite this, all groups vaccinated with the various emulsion formulations (with or without TLR agonists) provided protection against a lethal XBB.1 challenge. However, the weight loss data exhibited the same trend of neutralization potential that was seen against XBB.1 and many other variants, in which the INI-2002-containing groups achieved significantly better protection against weight loss compared to either AS03-like or Addavax adjuvanted groups.

There is reason to expect that T cells also contributed to disease prevention, particularly in the absence of high levels of neutralizing antibodies against a novel variant. A study of the correlates of protection in the rhesus macaque model demonstrated the importance of neutralizing antibodies, but also confirmed that CD8^+^ T cells play a role^18^. Therefore, a vaccine that boosts cross-reactive antibody titers and also promotes a Th1-skewed response, with the development of cytotoxic T lymphocytes, could provide an advantage in protection against antigenically diverse SARS-CoV-2 variants. The vaccines that include INI-2002 and/or INI-4001 emulsions showed a dramatic increase in Th1 and Th17 cell development coupled with a significant decrease in Th2 cells, as evidenced by the cytokine profile produced following T cell restimulation. While this occurred in all groups containing a TLR agonist, it should be noted that the TLR7/8 agonist INI-4001 was the most effective at promoting the development of a Th1 response, while the TLR4 agonist INI-2002 induced a mixed Th1/Th17 response. Antigen-specific CD8^+^ T cells were also detected by flow cytometry in the groups with TLR agonists. Not only did the cross-reactive antibody titers correlate with improved resistance to XBB.1, but we noted a similar correlation with the development of both Th1 and CD8^+^ T cell responses. The ability to add TLR agonists to existing emulsion-based adjuvants represents a way to improve both the humoral and cell-mediated immune response to optimize the result of vaccination and increase the breadth of immunity against antigenic variation.

To summarize, we have shown that emulsion-based vaccines can be significantly improved through the addition of a TLR agonist to expand protection over a vast antigenic space, even against antigenically distant Omicron subvariants like XBB.1. These adjuvants may allow a risk-averse subunit-based vaccine to provide protection equal or superior to mRNA or inactivated vaccines by eliciting both a high neutralizing antibody titer and robust cell-mediated immunity.

## Methods

### Antigens and adjuvants

Purified, recombinantly-expressed, ancestral SARS-CoV-2 Wuhan-Hu-1 trimerized spike ectodomain that had been stabilized through the introduction of two prolines and removal of the polybasic cleavage site (hereafter referred to as spike) was produced in Expi293F cells (Thermo Fisher Scientific) and purified as previously described^19–21^. Recombinant Wuhan-Hu-1 spike receptor binding domain (RBD) monomers were also produced through expression in Expi293F cells followed by purification.^21^ TLR4 agonist INI-2002^22^ and TLR7/8 agonist INI-4001^23^ were provided by Inimmune Corp (Missoula, MT, USA). An emulsion system containing 2.5% *v/v* squalene (MP Biomedicals, Irvine, CA, USA), 2.5% *v/v* α-tocopherol (TCI), and 0.96% *v/v* Tween-80 (Spectrum Chemical, New Brunswick, NJ, USA) in Dulbecco’s phosphate buffered saline (DPBS, pH 6.8), similar to Adjuvant System 03 (AS03™, GlaxoSmithKline), was used in this study. Since the emulsions used here were made outside of a current good manufacturing practice setting with research reagent grade materials, we refer to the emulsion as “AS03-like.” As an additional comparator, the commercially available equivalent of the squalene-based emulsion MF59™, AddaVax™ (Invivogen, San Diego, CA, USA) was used. All emulsion formulations were used at a concentration that delivered 180 μg of squalene per animal.

### Preparation and characterization of emulsions

Emulsions were prepared by thin-film rehydration technique followed by a combination of sonication and high-pressure homogenization as previously described^7^. First, a homogenous oil phase containing squalene, DL-α-tocopherol, polysorbate-80, and adjuvant (INI-4001 or INI-2002) was prepared by solubilization in isopropanol. The adjuvant was added to provide 10 μg for every 200 μg of squalene in the emulsions. A thin film was formed by evaporating the organic solvent using a Buchi rotary flash evaporator (New Castle, DE, USA). Both the oil phase and aqueous phase (DPBS at pH 6.8) were incubated at 50 °C, and the aqueous phase was added to the oil phase under bath sonication at 50 °C, followed by 15 cycles of ultra-acoustic focusing for 10 min with a Covaris (Woburn, MA, USA) S2 Ultrasonicator. The resulting crude emulsion was microfluidized by high-pressure homogenization using four passes at 25,000 PSI with a Microfluidics (Westwood, MA, USA) LV1. The emulsions were then sterile filtered using a 13 mm Millex-GV polyvinyldene difluoride (PVDF) filter with a pore size of 0.22 μm (MilliporeSigma, Burlington, MA, USA).

The particle size and polydispersity index of samples diluted 20-fold in water were measured using a Zetasizer Nano-ZS (Malvern Panalytical, Malvern, UK). The morphology of the emulsions was visualized using transmission electron microscopy performed by the Multiscale Microscopy Core at Oregon Health & Science University (OHSU)/FEI Living Lab and the OHSU Center for Spatial Systems Biomedicine. The samples were prepared by transfer to Quantifoil EM grids and freezing in liquid ethane using a Vitrobot prior to imaging. When necessary, the samples were diluted with the sample buffer. The samples were imaged using a FEI Talos Arctica system with a FEI Ceta 16 M CMOS camera (Thermo Fisher Scientific, Waltham, MA, USA). The images were processed using the Fiji distribution of ImageJ (NIH, Bethesda, MD, USA).

The final concentrations of the adjuvants in the formulations were determined using a Thermo Scientific Vanquish Flex UHPLC (Thermo Fisher Scientific, Waltham, MA, USA) equipped with a UV-visible and charged aerosol detector. A Waters (Millford, MA, USA) XSelect® CSH™ C18 2.1x 150 mm, 2.5 μm particle size column at 65 °C was used to elute the compounds using an acetonitrile and isopropyl alcohol gradient with 10 mM ammonium formate and 0.1% formic acid, at 0.3 ml/min.

Based on the concentrations of INI-4001 and INI-2002 in the emulsion, the appropriate amount to provide 10 μg of INI-4001 and/or INI-2002 per dose was mixed with the antigen to make the vaccine within an hour of injection. As the squalene itself has adjuvant activity, we added additional emulsion to adjust the final amount of squalene to 180 μg per dose. To also compare with another squalene-based emulsion adjuvant, the research-grade version of MF59™, Addavax™ (Invivogen) was used, also dosed to deliver 180 μg of squalene per animal.

### Vaccinations for immunogenicity studies

C57BL/6 J mice of 6–8 weeks in age were obtained from The Jackson Laboratory (Bar Harbor, ME) and allowed to recover for a minimum of five days prior to vaccination. Mice were housed in an Association for Assessment and Accreditation of Laboratory Animal Care (AAALAC) accredited facility and experiments were performed under animal use protocols approved by the University of Montana Institutional Animal Care and Use Committee. Vaccinations shown in Figure S1 used recombinant Wuhan-Hu-1 spike RBD monomers (3 μg/mouse) or Wuhan-Hu-1 spike trimerized ectodomain (1 μg/mouse). Doses were selected based upon antibody titers in previous antigen dose-response studies (not shown). These antigens were administered either alone in an aqueous solution or in combination with 10 μg/mouse aqueous forms of TLR4 agonist INI-2002 or TLR7/8 agonist INI-4001. Vaccinations shown in Figs. 2–5 used emulsions mixed with the Wuhan-Hu-1 spike trimerized ectodomain that had been thawed on ice and diluted to deliver 0.5 μg of spike protein, 180 μg of squalene, and 10 μg of INI-4001 and/or INI-2002 in the appropriate groups, per 50 μL volume. Mice were immunized twice by injection of 50 μL in the gastrocnemius muscle of one leg 21 days apart. Twenty-one days following the booster vaccination the mice were sacrificed for collection of blood, spleen, and the popliteal and inguinal draining lymph nodes (dLN) from the side of injection.

### Serum antibody enzyme-linked immunosorbent assay (ELISA)

Serum was isolated from blood collected at 21 days after booster immunization with Microtainer serum separator tubes (BD Biosciences) and stored at −20 °C until assayed. Serum was tested for anti-RBD IgG (Figure S1) or anti-spike IgG, IgG1, or IgG2a antibody titers by ELISA. Nunc Maxisorp 96-well plates (Thermo Fisher Scientific, Waltham, MA, USA) were coated overnight at room temperature with 0.25 μg/mL recombinant Wuhan-Hu-1 spike trimers in phosphate buffered saline (PBS), or 5 μg /mL Wuhan-Hu-1 RBD in PBS, then washed three times with PBS + 0.05% Tween-20 (PBS-T) and blocked with 200 μL enzyme immunoassay (EIA) buffer (1% bovine serum albumin, 0.1% Tween-20, and 5% heat-inactivated fetal bovine serum in PBS) for 1 h at 37 °C. Starting serum dilutions from 1:20 to 1:5000 were made in SuperBlock (Scytek Laboratories, Logan, UT, USA) according to the expected antibody response. After removal of EIA, an 8-point series of 3-fold serial dilutions in SuperBlock was made for each diluted serum sample, and allowed to bind for 2 h at 37 °C. Plates were washed three times with PBS-T, then incubated with goat anti-mouse IgG conjugated to horseradish peroxidase (IgG-HRP), IgG1-HRP, or IgG2c-HRP (Southern Biotech, Birmingham, AL, USA) diluted at 1:5000 in SuperBlock for 1 h at 37 °C. Plates were washed three times with PBS-T, then 100 μL per well of 3,3′,5,5′-tetramethylbenzidine (TMB) substrate was added for 20 min. Reactions were stopped by addition of 50 μL per well of 2 N sulfuric acid, and absorbance at 450 nm was measured with a SpectraMax 190 (Molecular Devices, San Jose, CA, USA) plate reader. The XLfit^®^ (IDBS, Boston, MA, USA) plug-in for Microsoft Excel was used to plot the data following subtraction of the background and determine the area under the curve (AUC).

Additional ELISAs were performed using the purified recombinant spike RBD from Wuhan-Hu-1, Omicron XBB.1, and SARS-CoV-1 (Urbani)^24^. As the full-length spike protein and RBD constructs are tagged with a polyhistidine (His)-tag, an irrelevant His-tagged influenza A virus neuraminidase (IAV NA-His) protein was utilized as a negative control to determine whether antibodies were elicited against this tag. Nunc Maxisorp 96-well plates were coated overnight at 4 °C with 2 μg/mL protein in 50 μL PBS per well. Plates were blocked for 1 hour at room temperature with 100 μL per well 3% non-fat milk (Life Technologies, Carlsbad, CA, USA) diluted in PBS-T. Sera was serially diluted 1:3 in PBS supplemented with 1% non-fat milk from a starting dilution of 1:120. A mouse anti-His monoclonal antibody (clone GT359, Sigma-Aldrich, St. Louis, MO, USA) was included as a positive control and an irrelevant, anti-influenza A HA stalk antibody, CR9114^25^, was used as a negative control. Both monoclonals were utilized at a starting concentration of 30 μg/mL and serially diluted 1:3. Following the block, plates were washed three times with PBS-T and 100 μL of sera dilutions were then added to each of the wells. 16 wells on each plate were left blank to serve as background controls. Sera dilutions were incubated on the plates for 2 h at room temperature before being removed prior to 3 washes with PBS-T. Plates were incubated with 100 μL of anti-mouse IgG conjugated to HRP (610-603-002, Rockland Immunochemicals, Limerick, PA, USA) diluted 1:3000 in 1% non-fat milk PBS-T for 1 h at room temperature, then again washed 3 times using PBS-T. Following washing, 100 μL of o-phenylenediamine dihydrochloride (OPD; Sigma-Aldrich) was added to each well and allowed to develop for 10 minutes before the reaction was stopped via the addition of 50 μL 3 M hydrochloric acid (HCl). Plates were read using a Synergy 4 plate reader (Biotek Instruments, Winooski, VT, USA) at an optical density of 490 nm. The data was exported into Prism 10 (GraphPad) and the area under the curve (AUC) was calculated using the average plus 3 standard deviations of the absorbance of the blank wells as the baseline.

### T cell cytokine measurements

The draining lymph nodes (dLN) and spleens were collected 21 days after booster immunization and manually disassociated into single-cell suspensions. Spleen cell suspensions underwent red blood cell (RBC) lysis by incubation in RBC lysis buffer (Biolegend, San Diego, CA, USA) for 5 minutes. Cells were counted using a Cellaca (Nexcelom, Lawrence, MA, USA) and resuspended in order to provide 5 × 10^6^ cells per well for spleens or 1.5 × 10^6^ per well for dLN cells, cultured individually except when too few cells were obtained, in which case cells from two animals were combined. Cells were cultured in a 96-well low evaporation plate (Nunclon™ Delta Surface Edge™, Thermo Fisher Scientific) in 200 μL complete RPMI, consisting of: Roswell Park Memorial Institute (RPMI) 1640 medium (Gibco/Thermo Fisher Scientific) containing 2 mM L-glutamine, 100 U/mL penicillin, 100 µg/mL streptomycin (Hyclone, Cytiva, Marlborough, MA, USA), 550 μM β-mercaptoethanol (Gibco/Thermo Fisher), and 10% heat-inactivated fetal bovine serum (Hyclone, Cytiva) for approximately 72 h. Cultures contained 10 μg/mL of spike protein to restimulate effector T cells. The plates were then centrifuged for five minutes at 400 *g* and a portion of the supernatant was collected and stored at −20 °C until analysis. The concentrations of interferon (IFN)γ, tumor necrosis factor (TNF)α, interleukin (IL)-17A and IL-5 in the supernatants were measured using a U-Plex kit from Meso Scale Discovery (Rockville, MD, USA) according to manufacturer’s instructions. Resulting concentrations were plotted using Prism 10 (GraphPad), and statistics provided by running one-way ANOVA analysis with correction for multiple comparisons by Tukey post-test.

### Flow cytometry

The dLN and spleen cell suspensions described above were cultured with 10 μg/mL Wuhan-Hu-1 spike trimers and 0.05 μg/mL overlapping Wuhan-Hu-1 spike peptide pool (GenScript RP30020, Piscataway, NJ, USA) for 18 h in complete RPMI (10% FBS, Pen/Strep/Glut, β-ME) in a tissue culture treated round-bottom 96-well culture plate. Brefeldin A (GolgiPlug™, BD Biosciences, Franklin Lakes, NJ, USA) was added for the last 6 hours of the culture to facilitate intracellular cytokine staining (ICS). Cells were collected by centrifugation at 400 *g* for five minutes at 4 °C. Cells were resuspended in 200 μL of DPBS containing fixable viability dye Ghost Dye 710™ (Tonbo, Cytek Biosciences, Fremont, CA, USA) diluted 1:200 for 30 min on ice protected from light. Cells were washed with 200 μL FACS buffer (FB, DPBS containing 2% FBS, 2 mM ethylenediaminetetraacetic acid, and 0.1% NaN_3_) and resuspended in 20 μL FB containing Fc Block™ (BD Biosciences, anti-mouse FcR monoclonal antibody (mAb) 2.4G2) at 0.05 μg/mL for 10 min protected from light on ice. Then 100 μL of a cocktail of fluorescently labeled antibodies against cell surface proteins in FB was added, and cells were incubated for 20 min protected from light on ice. This cocktail consisted of: anti-CD69-PE (H1.2F3, BioLegend 1:50), anti-CD44-PE-Cy7 (IM7, BioLegend, 1:100), anti-CD4a-APC-Cy7 (GK1.5, BioLegend 1:150), anti-CD8a-Brilliant Violet (BV)711 (53-6.7, BioLegend, 1:50) and anti-CD3e-BV421 (145-2C11, BD Biosciences, 1:50). After incubation, cells were washed twice with 200 μL FB. Cells were then fixed and permeabilized by resuspension in 100 μL Cytofix/Cytoperm buffer (BD Biosciences) for 20 min, followed by two washes with 200 μL PermWash. Cells were resuspended in 100 μL of a cocktail of fluorescently labeled antibodies against cytokines in PermWash buffer consisting of: anti-IFNγ-PE-CF594 (XMG1.2, BD Biosciences, 1:50), anti-IL-17A-PerCP-Cy5.5 (Invitrogen, Thermo Fisher Scientific, eBio17B7 1:50), and anti-IL-4-APC (11B11, Tonbo/Cytek, 1:50). After 30 min protected from light on ice, cells were washed twice with 200 μL PermWash buffer, followed by final resuspension in 150 μL per well of FB.

Cells were analyzed on a BD Biosciences LSRII, and data were analyzed using FlowJo™10.10.0 (BD Biosciences). Analysis included selection of the region known to contain lymphocytes by forward scatter and side scatter, doublet exclusion, and selection of viability dye negative (live) cells. Then samples were gated on CD3ε^+^CD4α^+^ or CD3ε^+^CD8α^+^ to select for T cells, and high expression of CD44 and CD69 for activation. This population was analyzed for production of IFNγ, IL-17A, and IL-4. These gates were placed with assistance from samples that had been stained for all markers and cytokines except the one being analyzed (fluorescence minus one control). The percentages of CD44^hi^CD69^hi^ T cells positive for cytokine were plotted using Prism 10 (GraphPad), and statistics were provided by running one-way ANOVA analysis with correction for multiple comparisons by Tukey post-test.

### Spike-angiotensin converting enzyme 2 (ACE2) interaction inhibition assay (SAIIA)

Serum collected from individual mice at 21 days after booster vaccination were tested for the ability to block the binding of recombinant soluble human ACE2 to immobilized recombinant spike proteins from several SARS-CoV-2 variants using V-Plex SARS-CoV-2/ACE2 kits from Meso Scale Discovery (Rockville, MD). Two panels were tested. Panel 23 contained Ancestral (Wuhan-Hu-1), Alpha (B.1.1.7), Beta (B.1.351), Delta (B.1.617.2), Gamma (P.1), and Omicron BA.1 (B.1.1.529). Panel 34 contained Ancestral (Wuhan-Hu-1), and Omicron variants B.1.1.529, BA.2.75, BA.5, BF.7, BN.1, BQ.1, BQ.1.1, XBB.1, and XBB.1.5. The assay was carried out according to manufacturer’s instructions using serum samples diluted 1:500 for panel 23 and 1:100 for panel 34 in the provided assay diluent. The percentage of binding that was blocked by the serum was determined by dividing the signal obtained in the presence of serum by the maximum signal obtained in the absence of serum, subtracting from 1.0, and multiplying by 100. Values were plotted using Prism 10 (GraphPad) and statistics provided by running One-way ANOVA analysis with correction for multiple comparisons by Tukey post-test.

### Microneutralization assay

All work with SARS-CoV-2 was performed in a biosafety level 3 (BSL3) laboratory. Vero.E6 cells expressing transmembrane protease, serine 2 (TMPRSS2 cat. 78081, BPS Biosciences, San Diego, CA, USA) were seeded in a 96-well cell culture plate (Corning; 3340) at a density of 2 × 10^4^ cells per well. Serum dilutions were prepared in 1× minimal essential medium (MEM; Gibco) supplemented with 2% fetal bovine serum (FBS). The irrelevant influenza mAb, CR9114, was included as a negative control at a starting concentration of 30 μg/mL and anti-viral remdesivir (Medkoo Bioscience, Morrisville, PA) was included as a positive control at a starting concentration of 100 μM. 24 h after cell seeding, virus was diluted to 1 × 10^4^ tissue culture infectious dose 50 (TCID_50_)/mL, and 80 μL of virus and 80 μL of sera dilution were combined and incubated together for 1 h at room temperature on a separate 96-well plate. After 1 h incubation, 120 μL of virus–antibody mixture was added to the cells for 1 h at 37 °C. After one hour, the virus-sera mixture was removed and 100 μL of each corresponding serum dilution was added to the wells. A volume of 100 μL of 1X MEM was also added, resulting in a total volume of 200 μL per well. Cells were incubated at 37 °C in a 5% CO_2_ incubator for 3 days prior to fixation with 10% paraformaldehyde (Polysciences) for 24 h. The paraformaldehyde was removed, cells were washed twice with PBS, and permeabilized via the addition of 150 μL of PBS supplemented with 0.1% Triton X-100 (Fisher). Cells were allowed to permeabilize at room temperature for 15 min before the permeabilization solution was removed. Cells were blocked for one hour with PBS supplemented with 3% non-fat milk (American Bio; catalog no. AB10109-01000), and then stained using the anti-nucleoprotein antibody 1C7 as previously described^21,26,27^.

### Infections

Animal procedures were performed according to a protocol approved by the Institutional Animal Care and Use Committee (IACUC) of the Icahn School of Medicine at Mount Sinai. To determine the 50% lethal dose (LD_50_) of wild-type SARS-CoV-2 (isolate USA-WA1/2020, BEI Resources NR-52281) and XBB.1 (isolate SARS-CoV-2/USA/NY-PV73997/2022, Mount Sinai Pathogen Surveillance Program), 6- to 8-week-old female, B6.Cg-Tg(K18-ACE2)2Prlmn/J mice (K18-hACE2, The Jackson Laboratory)^28^ were infected with serial dilutions of virus ranging from 10^5^ to 5 plaque forming units (PFU), diluted in 50 μL sterile phosphate-buffered saline (PBS).

For infection studies, 6–8-week-old female K18-hACE2 mice (*n* = 5 mice per group) from The Jackson Laboratory were vaccinated twice 21 days apart, as described above. Four weeks post-boost, blood was sampled from the mice prior to anesthetization via the intraperitoneal injection of 0.15 mg/kg ketamine and 0.03 mg/kg xylazine diluted in water for injection (WFI; Gibco). Following anesthesia, mice were intranasally infected with a 3xLD_50_ dose of either USA-WA1/2020 or XBB.1 SARS-CoV-2 diluted in 50 μL of sterile PBS. Weights were recorded over a period of 2 weeks and mice falling below 75% of their original body weight were humanely euthanized and scored as dead. Statistical analysis of body weight curves was done at each timepoint with Graphpad Prism, by doing unpaired t tests with Welch correction and correction for multiple comparisons by the Holm-Sidak method.

## Supplementary information

Supplementary data

## Data Availability

Data underlying this study can be found in ImmPort under identifier SDY2665.
