# Supplementary material for: Vaccination with ancestral SARS-CoV-2 spike adjuvanted with TLR agonists provides cross-protection against XBB.1
Source: Npj Viruses. 2024 Aug 6;2:28. doi: 10.1038/s44298-024-00038-0 (PMC11721079; doi:10.1038/s44298-024-00038-0)
Supplement: Supplementary file 1 — Supplementary data [file 44298_2024_38_MOESM1_ESM.pdf]

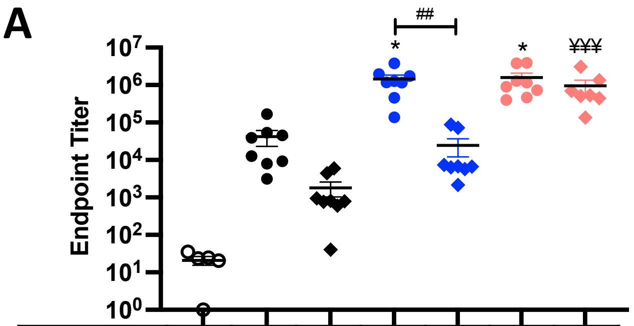

Spike (µg)	–	1	–	1	–	1	–
RBD (µg)	–	–	3	–	3	–	3
INI-4001 (µg)	–	–	–	10	10	–	–
INI-2002 (µg)	–	–	–	–	–	10	10

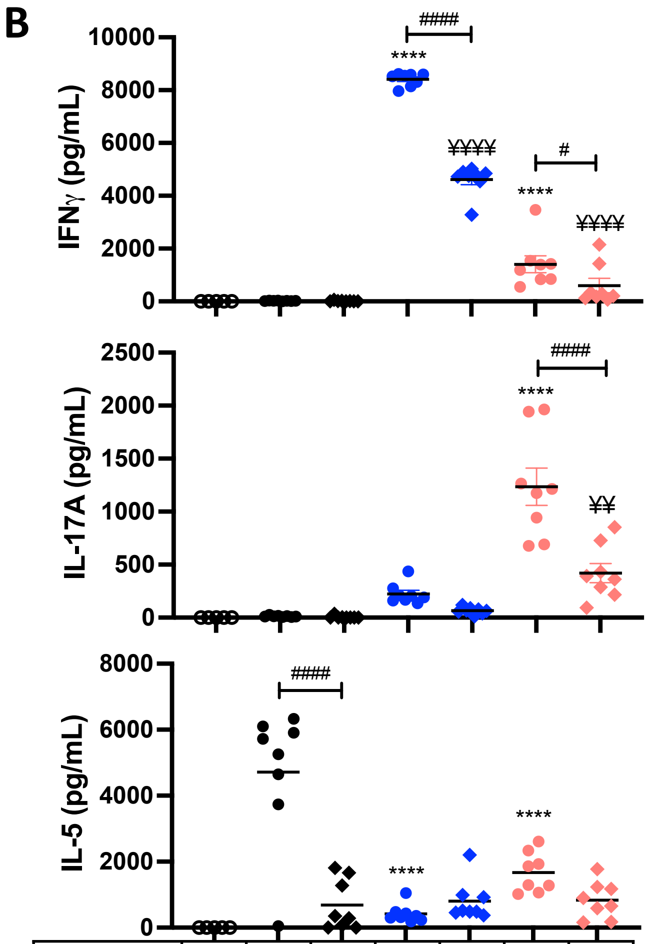

Spike (µg)	–	1	–	1	–	1	–
RBD (µg)	–	–	3	–	3	–	3
INI-4001 (µg)	–	–	–	10	10	–	–
INI-2002 (µg)	–	–	–	–	–	10	10

Supplementary Figure 1. Comparison of the immunogenicity of RBD and spike antigens. Mice were vaccinated 14 days apart with the indicated doses of antigen and adjuvant. Each symbol represents data from an individual animal. A) Serum from 14 days after 2nd vaccination was tested for RBD-specific IgG antibodies by ELISA. Comparisons were made with a Kruskal-Wallis test with Dunn's correction for multiple comparisons. B) Cell suspensions of draining lymph nodes collected 14 days after 2nd vaccination were cultured for 72h with both spike trimers and peptide pools. Supernatants were assayed for cytokines by multiplex ELISA. Comparisons were made by one-way ANOVA with Tukey correction for multiple comparisons. For both panels, * indicates significance versus the spike only group, ¥ versus the RBD only group, and # between indicated groups, $^{*/\#}p<0.05$, $^{**/¥¥/\#\#}p<0.01$, $^{¥¥¥}p<0.001$, $^{****/¥¥¥¥/\#\#\#\#}p<0.0001$

**IgG** Comparison	Spike only	Spike + AS03-like emul	Spike + AS03/ INI-2002	Spike + AS03/ INI-4001	Spike + AS03/ INI-2002/ INI-4001	Spike + Addavax
Spike only		0.0002	<0.0001	<0.0001	<0.0001	0.0134
Spike + AS03-like emul			0.0012	n.s.	0.0007	n.s.
Spike + AS03/ INI-2002				n.s.	n.s.	<0.0001
Spike + AS03/ INI-4001					n.s.	0.0299
Spike + AS03/ INI-2002/ INI-4001						<0.0001

**IgG1** Comparison	Spike only	Spike + AS03-like emul	Spike + AS03/ INI-2002	Spike + AS03/ INI-4001	Spike + AS03/ INI-2002/ INI-4001	Spike + Addavax
Spike only		<0.0001	n.s.	0.0250	n.s.	<0.0001
Spike + AS03-like emul			<0.0001	<0.0001	<0.0001.	n.s.
Spike + AS03/ INI-2002				n.s.	n.s.	<0.0001
Spike + AS03/ INI-4001					n.s.	<0.0001
Spike + AS03/ INI-2002/ INI-4001						<0.0001

**IgG2c** Comparison	Spike only	Spike + AS03-like emul	Spike + AS03/ INI-2002	Spike + AS03/ INI-4001	Spike + AS03/ INI-2002/ INI-4001	Spike + Addavax
Spike only		n.s.	<0.0001	<0.0001	<0.0001	n.s.
Spike + AS03-like emul			<0.0001	<0.0001	<0.0001	n.s.
Spike + AS03/ INI-2002				n.s.	n.s.	<0.0001
Spike + AS03/ INI-4001					0.0015	<0.0001
Spike + AS03/ INI-2002/ INI-4001						<0.0001

Supplementary Figure 2. Reporting of p values for differences shown to the be significant in the assays from Figure 3A, reporting the anti-spike serum antibody titers. A) Comparisons were made with a one-way ANOVA, with individual comparisons being calculated by the Tukey's multiple comparisons test. Significance was indicated by a p value of <0.05. Higher p values are reported as n.s. (not significant).

A

**Wuhan-1** Comparison	Spike only	Spike + AS03-like emul	Spike + AS03/ INI-2002	Spike + AS03/ INI-4001	Spike + AS03/ INI-2002/ INI-4001	Spike + Addavax
Spike only		<0.0001	<0.0001	<0.0001	<0.0001	<0.0001
Spike + AS03-like emul			0.0021	0.0070	0.0081	n.s.
Spike + AS03/ INI-2002				n.s.	n.s.	0.0004
Spike + AS03/ INI-4001					n.s.	0.0015
Spike + AS03/ INI-2002/ INI-4001						0.0017

**B.1.1.7** Comparison	Spike only	Spike + AS03-like emul	Spike + AS03/ INI-2002	Spike + AS03/ INI-4001	Spike + AS03/ INI-2002/ INI-4001	Spike + Addavax
Spike only		<0.0001	<0.0001	<0.0001	<0.0001	<0.0001
Spike + AS03-like emul			0.0044	0.0348	0.0201	n.s.
Spike + AS03/ INI-2002				n.s.	n.s.	0.0004
Spike + AS03/ INI-4001					n.s.	0.0015
Spike + AS03/ INI-2002/ INI-4001						0.0017

**B.1.617.2** Comparison	Spike only	Spike + AS03-like emul	Spike + AS03/ INI-2002	Spike + AS03/ INI-4001	Spike + AS03/ INI-2002/ INI-4001	Spike + Addavax
Spike only		<0.0001	<0.0001	<0.0001	<0.0001	<0.0001
Spike + AS03-like emul			<0.0001	0.0006	0.0012	n.s.
Spike + AS03/ INI-2002				n.s.	n.s.	0.0002
Spike + AS03/ INI-4001					n.s.	0.0012
Spike + AS03/ INI-2002/ INI-4001						0.0023

**B.1.351** Comparison	Spike only	Spike + AS03-like emul	Spike + AS03/ INI-2002	Spike + AS03/ INI-4001	Spike + AS03/ INI-2002/ INI-4001	Spike + Addavax
Spike only		<0.0001	<0.0001	<0.0001	<0.0001	<0.0001
Spike + AS03-like emul			0.0021	0.0070	0.0081	n.s.
Spike + AS03/ INI-2002				n.s.	n.s.	0.0004
Spike + AS03/ INI-4001					n.s.	0.0015
Spike + AS03/ INI-2002/ INI-4001						0.0017

**P.1** Comparison	Spike only	Spike + AS03-like emul	Spike + AS03/ INI-2002	Spike + AS03/ INI-4001	Spike + AS03/ INI-2002/ INI-4001	Spike + Addavax
Spike only		n.s.	<0.0001	0.0002	<0.0001	0.0211
Spike + AS03-like emul			n.s.	0.0002	0.0038	n.s.
Spike + AS03/ INI-2002				n.s.	n.s.	0.0022
Spike + AS03/ INI-4001					n.s.	n.s.
Spike + AS03/ INI-2002/ INI-4001						0.0383

**B.1.1.529** Comparison	Spike only	Spike + AS03-like emul	Spike + AS03/ INI-2002	Spike + AS03/ INI-4001	Spike + AS03/ INI-2002/ INI-4001	Spike + Addavax
Spike only		n.s.	0.0003	n.s.	n.s.	n.s.
Spike + AS03-like emul			0.0006	n.s.	n.s.	n.s.
Spike + AS03/ INI-2002				0.0021	n.s.	0.0003
Spike + AS03/ INI-4001					n.s.	n.s.
Spike + AS03/ INI-2002/ INI-4001						n.s.

B

**Wuhan-1** Comparison	Spike only	Spike + AS03-like emul	Spike + AS03/ INI-2002	Spike + AS03/ INI-4001	Spike + AS03/ INI-2002/ INI-4001	Spike + Addavax
Spike only		<0.0001	<0.0001	<0.0001	<0.0001	<0.0001
Spike + AS03-like emul			n.s.	n.s.	n.s.	n.s.
Spike + AS03/ INI-2002				n.s.	n.s.	n.s.
Spike + AS03/ INI-4001					n.s.	n.s.
Spike + AS03/ INI-2002/ INI-4001						n.s.

**B.1.1.529** Comparison	Spike only	Spike + AS03-like emul	Spike + AS03/ INI-2002	Spike + AS03/ INI-4001	Spike + AS03/ INI-2002/ INI-4001	Spike + Addavax
Spike only		<0.0001	<0.0001	<0.0001	<0.0001	<0.0001
Spike + AS03-like emul			0.0024	n.s.	n.s.	n.s.
Spike + AS03/ INI-2002				0.0021	n.s.	0.0003
Spike + AS03/ INI-4001					n.s.	n.s.
Spike + AS03/ INI-2002/ INI-4001						0.0471

**XBB.1** Comparison	Spike only	Spike + AS03-like emul	Spike + AS03/ INI-2002	Spike + AS03/ INI-4001	Spike + AS03/ INI-2002/ INI-4001	Spike + Addavax
Spike only		<0.0001	<0.0001	<0.0001	<0.0001	<0.0001
Spike + AS03-like emul			0.0454	n.s.	n.s.	n.s.
Spike + AS03/ INI-2002				n.s.	n.s.	0.0004
Spike + AS03/ INI-4001					n.s.	n.s.
Spike + AS03/ INI-2002/ INI-4001						n.s.

Supplementary Figure 3. Reporting of p values for differences shown to the be significant in Figure 4, in which the serum neutralization potential was reported by the ability of serum to block huACE2 binding to the spike protein from the indicated variant. A) Significance in Figure 4B. Comparisons were made with a one-way ANOVA, with individual comparisons being calculated by the Tukey's multiple comparisons test. The serum in these assays was used at a 1:500 dilution. B) Significance in Figure 4C. Comparisons were made as in A but using a 1:100 serum dilution. For all assays, significance was indicated by a p value of <0.05. Higher p values are indicated as n.s. (not significant).

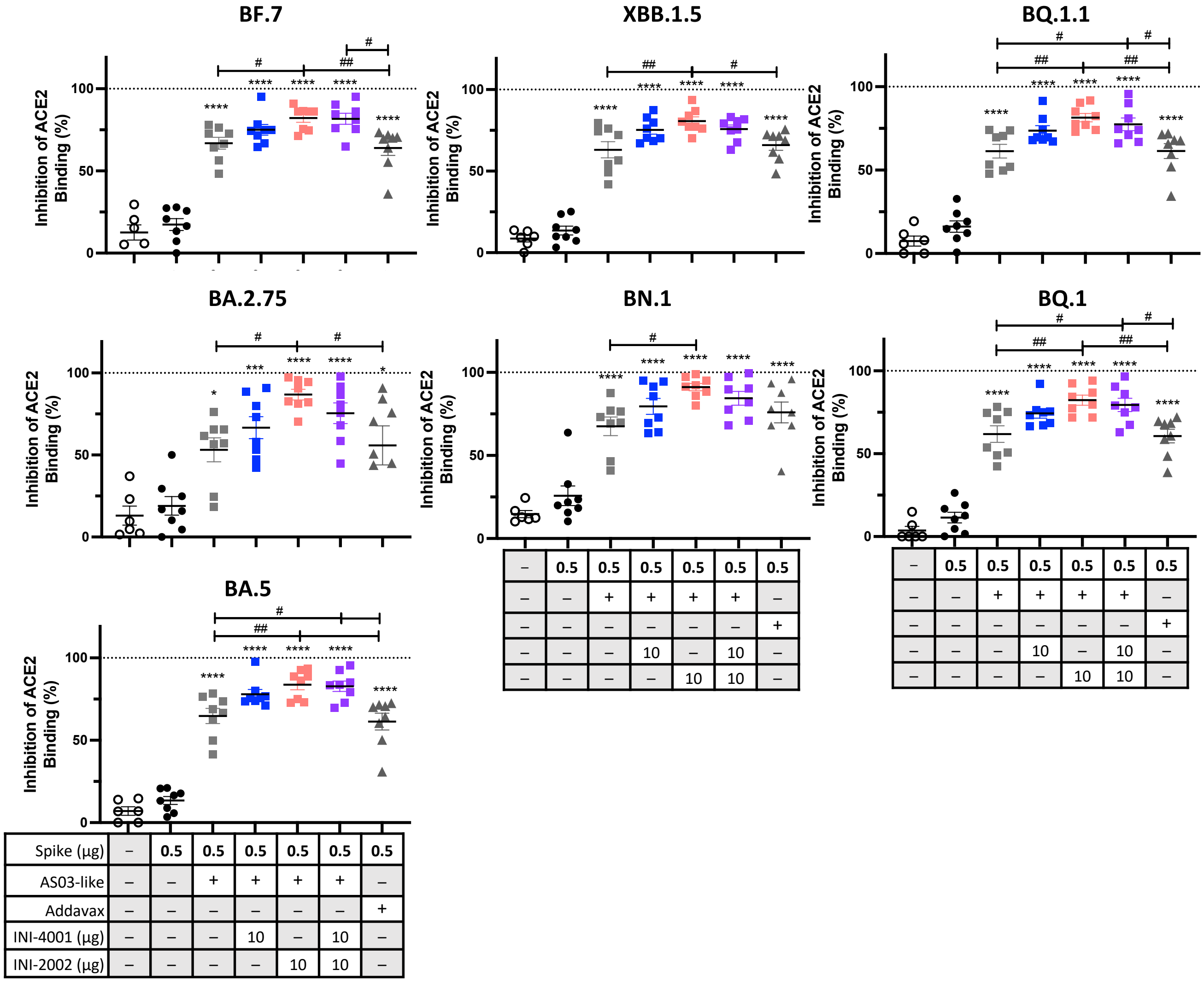

Supplementary Figure 4. Neutralization potential of serum from vaccinated mice (related to Figure 4). Serum collected 21 days after booster vaccination was tested for the ability to block the binding of soluble human ACE2 to recombinant spike trimers from the indicated Omicron strains by multiplexed competitive ELISA. Serum was diluted 1:100. Differences between groups were analyzed by one-way ANOVA with multiple comparisons by Tukey post-test. Asterisks denote significant differences versus unadjuvanted Spike antigen and #'s indicate differences between other indicated groups. $^{*/\#}$p<0.05, $^{\#\#}$p<0.01, $^{***/\#\#\#}$p<0.001, $^{****/\#\#\#\#}$p<0.0001

**BF.7** Comparison	Spike only	Spike + AS03-like emul	Spike + AS03/ INI-2002	Spike + AS03/ INI-4001	Spike + AS03/ INI-2002/ INI-4001	Spike + Addavax
Spike only		<0.0001	<0.0001	<0.0001	<0.0001	<0.0001
Spike + AS03-like emul			0.0407	0.0070	n.s.	n.s.
Spike + AS03/ INI-2002				n.s.	n.s.	0.0085
Spike + AS03/ INI-4001					n.s.	n.s.
Spike + AS03/ INI-2002/ INI-4001						0.0108

**XBB.1.5** Comparison	Spike only	Spike + AS03-like emul	Spike + AS03/ INI-2002	Spike + AS03/ INI-4001	Spike + AS03/ INI-2002/ INI-4001	Spike + Addavax
Spike only		<0.0001	<0.0001	<0.0001	<0.0001	<0.0001
Spike + AS03-like emul			0.0052	n.s.	n.s.	n.s.
Spike + AS03/ INI-2002				n.s.	n.s.	0.0290
Spike + AS03/ INI-4001					n.s.	0.0015
Spike + AS03/ INI-2002/ INI-4001						0.0017

**BQ.1.1** Comparison	Spike only	Spike + AS03-like emul	Spike + AS03/ INI-2002	Spike + AS03/ INI-4001	Spike + AS03/ INI-2002/ INI-4001	Spike + Addavax
Spike only		<0.0001	<0.0001	<0.0001	<0.0001	<0.0001
Spike + AS03-like emul			0.0039	n.s.	0.0331	n.s.
Spike + AS03/ INI-2002				n.s.	n.s.	0.0040
Spike + AS03/ INI-4001					n.s.	n.s.
Spike + AS03/ INI-2002/ INI-4001						0.0340

**BA.2.75** Comparison	Spike only	Spike + AS03-like emul	Spike + AS03/ INI-2002	Spike + AS03/ INI-4001	Spike + AS03/ INI-2002/ INI-4001	Spike + Addavax
Spike only		0.0221	0.0005	<0.0001	<0.0001	0.010
Spike + AS03-like emul			0.0254	n.s.	n.s.	n.s.
Spike + AS03/ INI-2002				n.s.	n.s.	0.0486
Spike + AS03/ INI-4001					n.s.	n.s.
Spike + AS03/ INI-2002/ INI-4001						n.s.

**BN.1** Comparison	Spike only	Spike + AS03-like emul	Spike + AS03/ INI-2002	Spike + AS03/ INI-4001	Spike + AS03/ INI-2002/ INI-4001	Spike + Addavax
Spike only		<0.0001	<0.0001	<0.0001	<0.0001	<0.0001
Spike + AS03-like emul			0.0210	n.s.	n.s.	n.s.
Spike + AS03/ INI-2002				n.s.	n.s.	n.s.
Spike + AS03/ INI-4001					n.s.	n.s.
Spike + AS03/ INI-2002/ INI-4001						n.s.

**BQ.1** Comparison	Spike only	Spike + AS03-like emul	Spike + AS03/ INI-2002	Spike + AS03/ INI-4001	Spike + AS03/ INI-2002/ INI-4001	Spike + Addavax
Spike only		<0.0001	<0.0001	<0.0001	<0.0001	<0.0001
Spike + AS03-like emul			0.0055	n.s.	0.0236	n.s.
Spike + AS03/ INI-2002				n.s.	n.s.	0.0029
Spike + AS03/ INI-4001					n.s.	n.s.
Spike + AS03/ INI-2002/ INI-4001						0.0132

**BA.5** Comparison	Spike only	Spike + AS03-like emul	Spike + AS03/ INI-2002	Spike + AS03/ INI-4001	Spike + AS03/ INI-2002/ INI-4001	Spike + Addavax
Spike only		<0.0001	<0.0001	<0.0001	<0.0001	<0.0001
Spike + AS03-like emul			0.0083	n.s.	0.0142	n.s.
Spike + AS03/ INI-2002				n.s.	n.s.	0.0012
Spike + AS03/ INI-4001					n.s.	0.0300
Spike + AS03/ INI-2002/ INI-4001						0.0022

Supplementary Figure 5. Reporting of p values for differences shown to the be significant in the assays from Figure S4, in which the serum neutralization potential was reported by the ability of serum to block huACE2 binding to the spike protein from the indicated Omicron variants. A) Comparisons were made with a one-way ANOVA, with individual comparisons being calculated by the Tukey's multiple comparisons test. The serum in these assays was used at a 1:100 dilution. Significance was indicated by a p value of <0.05. Higher p values are indicated as n.s. (not significant).
